# Effect of cardiac resynchronisation therapy in dilated cardiomyopathy

**DOI:** 10.1007/s12471-021-01548-9

**Published:** 2021-02-23

**Authors:** S. Bouwmeester, L. X. van Nunen

**Affiliations:** Department of Cardiology, Catharina Hospital Eindhoven, Eindhoven, The Netherlands

A 48-year-old woman presented with progressive exertional dyspnoea, orthopnoea and weight gain. Physical examination revealed bilateral rales, a third heart sound, a holosystolic murmur over the apex, abdominal and peripheral oedema.

Electrocardiogram showed left bundle branch block (Fig. 1 panel a). Heart size was markedly increased on chest X‑ray (Fig. 1 panel b). On transthoracic echocardiography left ventricular ejection fraction was severely depressed (LVEF 16%; video 1) with severe secondary mitral regurgitation. This was corroborated by cardiac magnetic resonance imaging (LVEF 18%). Coronary artery disease was excluded on coronary computed tomography. The patient was diagnosed with dilated cardiomyopathy and treated with optimal medical therapy.

**Fig. 1:**
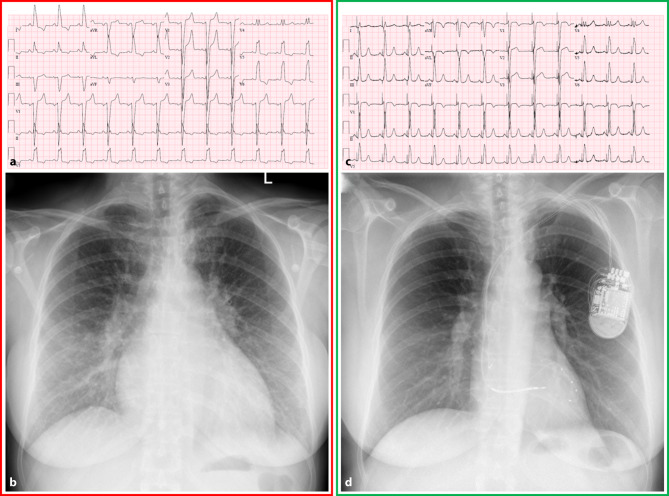
Electrocardiogram and chest X‑ray before (panel **a** and panel **b**) and after (panel **c** and **d**) cardiac resynchronisation therapy

Cardiac resynchronisation therapy was initiated under which QRS duration decreased significantly (Fig. 1 panel c), as well as a marked reduction in heart size on chest X‑ray and transthoracic echocardiography (Fig. 1 panel d; video 2). The patient rapidly recovered and shows no signs or symptoms of heart failure.

## Supplementary Information

**Video 1**: Transthoracic echocardiogram showed LV dyssynchrony with severely depressed ejection fraction.

**Video 2**: Transthoracic echocardiogram showed improved LV synchrony and ejection fraction after cardiac resynchronization therapy.

